# Association between pterygium, sun exposure, and serum 25-hydroxyvitamin in a nationally representative sample of Korean adults

**DOI:** 10.1186/s12944-018-0902-6

**Published:** 2018-11-20

**Authors:** Yoon Hong Chun, Ji-Sun Paik, Ju Heun Oh, Hyun-Seung Kim, Kyung-Sun Na

**Affiliations:** 1 Department of Pediatrics, Incheon St. Mary’s Hospital, College of Medicine, The Catholic University of Korea, Incheon, South Korea; 2 Department of Ophthalmology, Seoul St. Mary’s Hospital, College of Medicine, The Catholic University of Korea, Seoul, South Korea; 3 Department of Ophthalmology, Yeouido St. Mary’s Hospital, College of Medicine, The Catholic University of Korea, Seoul, South Korea

**Keywords:** Sun exposure, Serum 25-hydroxyvitamin D, Pterygium, Large population-based study, Epidemiology

## Abstract

**Purpose:**

Ultraviolet-B (UVB) light exposure is the major risk factor for developing a pterygium, and serum 25-hydroxyvitamin D (25(OH)D) level is an objective measure of UVB light exposure. In this study, we investigated the association between pterygium, sun exposure, and serum 25(OH)D.

**Methods:**

This population-based, cross-sectional study comprised 12,258 adults (aged ≥19 years) participating in the fifth annual Korea National Health and Nutrition Examination Survey from 2010 to 2012. The enrolled subjects underwent interviews, clinical examinations, and laboratory investigations. The serum 25(OH)D levels were measured, and pterygium was examined by using a slit lamp. We used three adjusted logistic regression models and selected covariates as potential confounders.

**Results:**

The overall prevalence of pterygium was 7.09, and 53.1% of these subjects were women. The prevalence of pterygium was higher in elderly subjects and those who lived at low latitudes. In multivariate analysis with adjustment for confounding factors, subjects with a serum 25(OH)D level > 30 ng/mL, 25–30 ng/mL, and 15–20 ng/mL had an odds ratio (OR) (95% confidence interval [CI]) of 1.565 (1.035–2.366), 1.545 (1.086–2.198), 1.8 (1.358–2.386), and 1.535 (1.216–1.938), respectively, compared to those with a serum 25(OH)D level < 15 ng/mL. Subjects with a daily sun exposure > 5 h had an OR (95% CI) of 1.761 (1.395–2.223) compared to subjects with a daily sun exposure < 2 h.

**Conclusion:**

The present study provides epidemiological evidence of an association of daily sun exposure and serum 25(OH)D levels with pterygium in a representative Korean population.

## Introduction

A pterygium is a fibrovascular growth of the conjunctiva, commonly encroaching onto the cornea [1]. Although usually small and benign in nature, pterygia are common; cause considerable irritation, astigmatism, and cosmetic concerns; and often recur after surgical removal [2, 3]. Altered limbal stem cells play a key role in the development of pterygium, and histopathological studies have revealed the role of chronic inflammation in its pathogenesis [4, 5].

Although the pathogenesis of pterygium formation remains unclear, one of the major demographic characteristics of pterygium is its strong link with geographical latitude, thought to be related to variations in ultraviolet-B (UVB) light exposure [6]. Epidemiological studies have revealed that the prevalence of pterygium is inversely related to latitude, and that it is greater among outdoor than indoor workers [7–9]. However, in previous studies, relatively subjective measurements of sunlight exposure with UVB irradiation were made based on surveys of outdoor job occupations, amount of leisure-time outdoor activities, and geographic location of residency [4, 10, 11]. Although sun exposure has been accepted as a risk factor for pterygium, there is no objective diagnostic tool to measure the total amount of sun exposure of an individual.

Vitamin D is a multifactorial hormone that is now known to play a significant role in a variety of biological functions, including immune regulation, proliferation, differentiation, apoptosis, and angiogenesis, in addition to its traditional role in regulating calcium homeostasis [12, 13]. Moreover, because many tissues in the eye are able to respond to vitamin D [14, 15], vitamin D levels influence the development of a wide range of eye pathologies, such as myopia, age-related macular degeneration, and diabetic retinopathy [16–18]. Considering that vitamin D is synthesized endogenously from exposure to sunlight, it may serve as a biomarker of cumulative UVB exposure [19]. Considering the fact that vitamin D production is primarily and fundamentally influenced by UVB exposure [19], we postulated that serum 25-hydroxyvitamin D (25(OH)D) levels might represent a surrogate marker for the exposure to UVB light, and could therefore be used for analysis of the association between UVB exposure and pterygium.

Very recently, some studies have suggested a possible association of vitamin D and pterygium, which is against the general mechanism that vitamin D has anti-neovascular and anti-inflammatory properties. In this study, we further analyzed the association between sun exposure, serum 25(OH)D, and pterygium in varied age groups, occupations, geographical latitudes, and blepharoptosis status (which may affect ocular UVB exposure), to provide more clues on the association between the blood levels of 25(OH)D and pterygium in a large representative sample of the Korean general population.

## Methods

### Study population

This population-based study collected data from the fifth (2010–2012) Korea National Health and Nutrition Examination Survey (KNHANES). The KNHANES comprises both independent and homogeneous annual rolling samples reported from South Korea. The survey aimed to assess the health and nutritional status of the stratified multistage probability samples of Korean households representing the noninstitutionalized civilian population. The KNHANES comprised a health interview survey, a health examination survey, and a nutrition survey.

The study design followed the standards of the Declaration of Helsinki for biomedical research, and the protocols for this study were approved by the Institutional Review Board of the Catholic University of Korea (XC17ZCDI0066). Informed consent was obtained from all study participants.

### Data collection

Demographic variables including age, sex, area of residence, education, income, smoking, alcohol drinking, regular exercise, and sun exposure were collected from the health interview survey. Occupation was categorized according to the fields of professional, office workers, service industry, agriculture/forestry/fishery, technician, laborer, or none (including housewife and student). The area of residence was categorized as urban and rural. Among the 16 districts of South Korea, eight major cities (Seoul, Gyeonggi, Busan, Daegu, Incheon, Gwangju, Daejeoun, and Ulsan) were grouped as urban areas, and the other provinces (Gangwon, Chungbuk, Chungnam, Jeonbuk, Jeonnam, Gyeongbuk, Gyeongnam, and Jeju) were grouped as rural areas. Education was categorized into high school education of ≤12 years or higher education. Monthly household income was categorized into the lowest or the three highest quartiles of income. Smoking status was divided into current smokers and non-smokers (including ex-smokers), and alcohol consumption status was divided into heavy drinkers and moderate drinkers to non-drinkers. Regular exercise was defined as walking for > 30 min at once, at least 5 times a week. Sunlight exposure was categorized as an average of < 2, 2–5, or > 5 h of sunlight per day.

Blood pressure was measured three times after 5 min of rest, and the average of the second and third measurements was considered as the final blood pressure. Venous blood samples were taken from the participants after fasting for at least 8 h. Fasting plasma glucose was measured by enzymatic methods using a Hitachi Automatic Analyzer 7600 (Hitachi, Tokyo, Japan). The serum 25(OH)D was measured using a radioimmunoassay kit (DiaSorin Inc., Stillwater, MN, USA) with a gamma counter (1470 WJZARD; Perkin-Elmer, Finland). Details of the 25(OH)D analysis have been reported previously [20]. Hypertension was defined as systolic blood pressure ≥ 140 mmHg, diastolic blood pressure ≥ 90 mmHg, or taking medication for the treatment of hypertension. Diabetes mellitus was defined as fasting blood glucose ≥126 mg/dL, being diagnosed as such by health care professionals, or taking insulin or antidiabetic medications.

Height, weight, and waist circumference (WC) were measured by specially trained examiners using standard anthropometric equipment. In this study, general obesity was defined as a body mass index (BMI) ≥25 kg/m^2^, and abdominal obesity was defined as a WC ≥90 cm for men and ≥ 80 cm for women, according to the criteria for obesity established by the West-Pacific region of the World Health Organization [21].

All ophthalmologic examinations were conducted by ophthalmologists using a slit lamp (Haag-Streit, Koeniz, Switzerland). A pterygium was defined as a radially oriented fibrovascular lesion crossing over the nasal or temporal limbus. Grading was based on the visibility of the underlying episcleral blood vessels: an atrophic pterygium was defined as a pterygium that allowed the clear discernment of the episcleral vessels, while a flesh-type pterygium was defined as a thick pterygium that did not allow the visualization of the episcleral vessels. All other pterygia that did not meet the definitions of these two categories were listed as an intermediate type. We defined a pterygium subject as a subject with a pterygium in at least one eye; in participants with pterygia in both eyes, we evaluated the most severe lesion. To measure the length of a pterygium, we measured the longest horizontal length from the limbus using horizontal slit illumination. Slit-lamp examinations were also performed to check for the presence of cataracts. Participants were defined as having a cataract if they had a nuclear, cortical, or posterior subcapsular cataract in at least one eye. Aphakia or pseudophakia were also documented but were excluded from the simple linear analysis.

### Statistical analysis

All statistical analyses were performed using SAS software (version 9.3; SAS Institute, Inc., Cary, NC, USA) and a survey procedure using sampling weights, defined by the KNHANES, to provide nationally representative estimates, adjusted for the survey year to minimize the variations between survey years. The statistical analyses were performed to compare the demographic characteristics of the study participants by pterygium status, as well as to compare the serum 25(OH)D levels and daily sun exposure by pterygium recurrence and morphology. All data are presented as proportions (standard error) for categorical variables and means ± standard errors for continuous variables.

Logistic regression analysis was performed to estimate the association of daily sun exposure, serum 25(OH)D level, and pterygium prevalence, and to confirm the association of serum 25(OH)D level and pterygium prevalence, which has been reported previously. The odds ratios (ORs) and 95% confidence intervals (CIs) were calculated using 3 different models. Model 1 was adjusted for age and exercise; model 2 was adjusted for age, exercise, BMI, smoking status, and alcohol consumption status; and model 3 was further adjusted for diabetes mellitus, metabolic syndrome, hypertension, and psychological stress. The adjusted factors were selected from univariate analysis and previous articles.

## Results

**Fig. 1:**
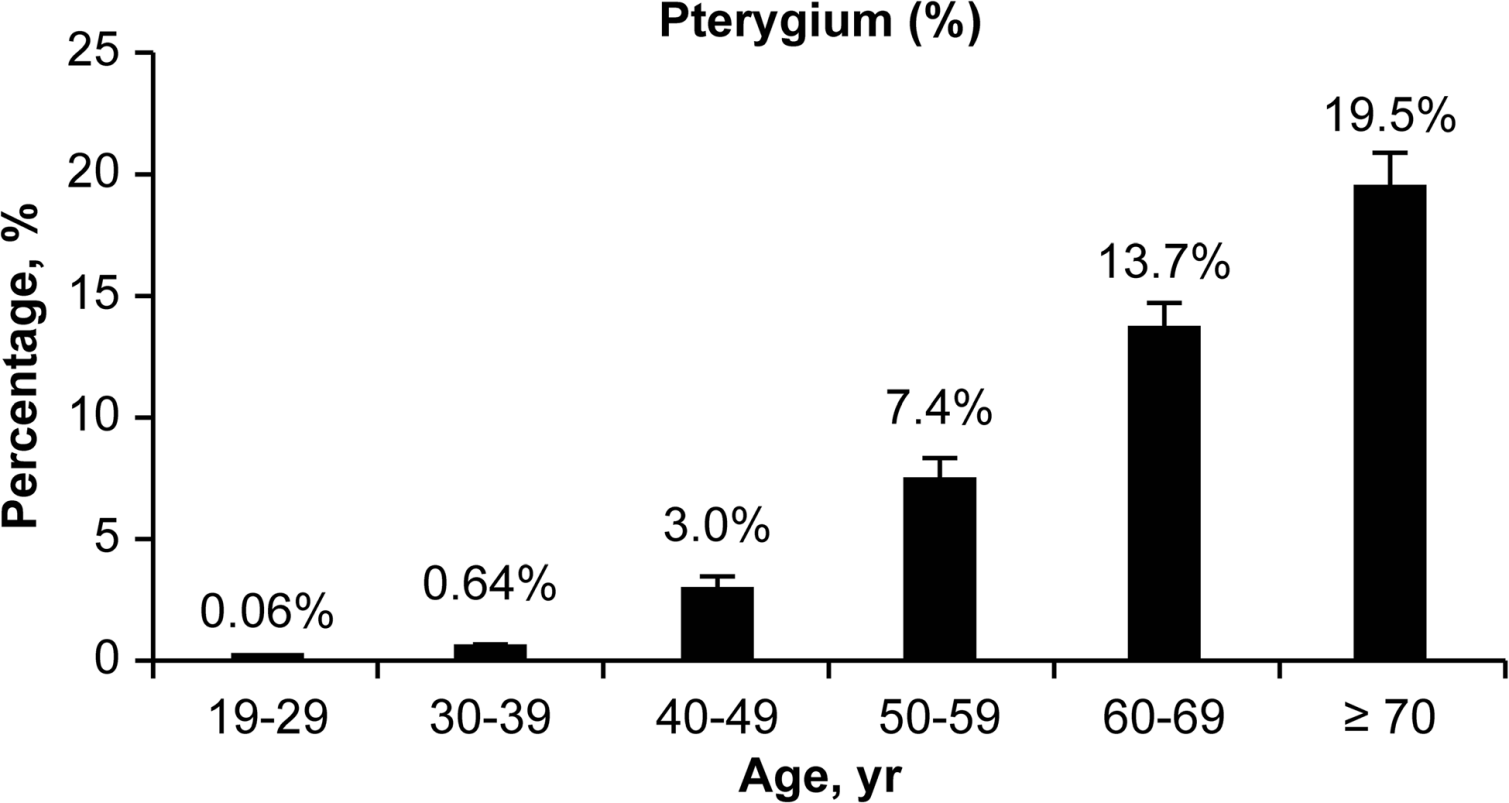
Prevalence of pterygium according to age

In the current study, 17,476 individuals for whom serum 25(OH)D levels were obtained were selected. Of these, 4170 subjects aged < 19 years and 1048 subjects with missing data were excluded. Finally, 12,258 participants aged ≥19 years were included in the analysis because this was the age group for which serum analyses were performed. The overall prevalence of pterygium was 7.09, and 53.1% (weighted percentage) of these subjects were women. The prevalence of pterygium was found to increase along with age (Fig. 1).

All participants were Asian, and the iris color was brown to dark brown. Among subjects aged ≥19 years, the prevalence of pterygium in at least one eye was 7.08%. Among the subjects with pterygium, 71.2% showed no recurrence, while 28.8% showed recurrence > 2 times. Regarding the morphology of pterygia, 55.9% were atrophic, 35.5% were intermediate, and 8.6% were flesh-type (data not shown).

Table 1 shows the demographic characteristics of subjects with and without pterygium. The mean age of subjects with and without pterygium was 44.3 ± 0.3 years and 63.2 ± 0.36 years, respectively (*P* < 0.001). Subjects with pterygium had larger WC, higher serum 25(OH)D level, lower education, and lower income than subjects without pterygium (*P* < 0.001). The prevalence of current smokers, cataract, blepharoptosis, diabetes, hypertension, and longer sun exposure was higher among subjects with pterygium than among those without pterygium (*P* < 0.001).

**Table 1:** Clinical characteristics of the study participants according to the presence of pterygium in the Korean population (n = 12,258)

	Pterygium		*P*
	No	Yes	
	*n* = 11,389	*n* = 869	
Age, years	44.3 ± 0.3	63.2 ± 0.6	< 0.001
Sex, % men	49.3 (0.5)	46.9 (2)	0.23
BMI, kg/m^2^	23.61 ± 0.05	23.9 ± 0.1	0.11
WC, cm	80.9 ± 0.2	83.5 ± 0.4	< 0.001
Serum 25(OH)D concentration, ng/mL	17.4 ± 0.2	19.7 ± 0.3	< 0.001
Education, % of ≥12 years	72.9 (0.8)	26.2 (2)	< 0.001
Income, % of lowest Q1	15.7 (0.6)	37.4 (2.1)	< 0.001
Current smoker, %	24.5 (0.6)	17.3 (1.6)	< 0.001
Heavy drinker, %	10.5 (0.4)	8.2 (1.4)	0.13
Regular exercise, %	20.8 (0.6)	22.8 (1.9)	0.25
Occupation, %	64.9 (0.6)	60.9 (2.4)	0.08
Cataract, %	22.7 (0.9)	61.1 (2.7)	< 0.001
Blepharoptosis, %	7.4 (0.5)	17.3 (1.8)	< 0.001
Metabolic syndrome, %	24.5 (0.5)	44.4 (2.4)	< 0.001
Diabetes, %	8.2 (0.3)	14.5 (1.7)	< 0.001
Hypertension, %	26.4 (0.6)	51.1 (2.3)	< 0.001
Daily sun exposure, %			< 0.001
< 2 h	60.7 (0.9)	46.8 (2.6)	
2–5 h	25.9 (0.8)	24 (2)	
> 5 h	13.4 (0.7)	29.3 (2.6)	

Data are presented as the weighted mean ± standard error or weighted percent (standard error)

*BMI* = body mass index, *WC* = waist circumference, *25(OH)D* = 25-hydroxyvitamin D

Table 2 shows the weighted percentages of individuals with pterygium for various serum 25(OH)D levels, daily sun exposures, and occupations. Similar trends are present in subjects with and without pterygium, yet there is a clear difference across the levels. The percentage of subjects with serum 25(OH)D level of 20–25 ng/mL, 25–30 ng/mL, and > 30 ng/mL increased in the pterygium group compare to the no-pterygium group. The percentage of subjects with daily sun exposure > 5 h was higher in the pterygium group compared to the no-pterygium group (the percentage of subjects without pterygium was 60.7% in the group with < 2 h of sun exposure and 13.4% in the group with > 5 h of sun exposure). The distribution of subjects with various serum 25(OH)D levels or daily sun exposure was not different in subjects with pterygium according to the recurrence and morphology of the pterygium. The percentage of subjects who had an occupation related to agriculture, forestry, or fishery was higher in the pterygium group than in the no-pterygium group.

**Table 2:** Serum 25(OH)D concentration, daily sun exposure, and occupation according to the recurrence and morphology of pterygium in the Korean population (n = 12,258)

	No pterygium	Recurrence		*P*	No pterygium	Morphology			*P*
		No recurrence	Recurrence			Atrophic	Intermediate	Flesh	
Serum 25(OH)D concentration, ng/mL				<.001					< 0.001
< 15	41.4 (1.1)	27.8 (2.4)	28.7 (3.3)		41.4 (1.1)	25.2 (2.5)	32 (3.7)	30.2 (7.2)	
15–20	31.3 (0.7)	30.7 (2.4)	33.6 (3.5)		31.3 (0.7)	34.3 (2.5)	26.4 (3.2)	34 (7.2)	
20–25	16.5 (0.6)	24.1 (2.3)	24.3 (3.6)		16.5 (0.6)	24.6 (2.8)	22.8 (2.6)	26.5 (6.8)	
25–30	7 (0.4)	11.2 (1.7)	8.3 (2.3)		7 (0.4)	10.5 (2)	11.1 (2.5)	6.1 (3.2)	
> 30	3.8 (0.4)	6.3 (1.3)	5.1 (1.3)		3.8 (0.4)	5.3 (1.3)	7.6 (1.9)	3.2 (1.9)	
Daily sun exposure, hours				< 0.001					< 0.001
< 2	60.7 (0.9)	50.3 (3)	38.1 (4.2)		60.7 (0.9)	52.1 (3.1)	45.5 (3.9)	16.8 (4.9)	
2–5	25.9 (0.8)	21.5 (2.2)	30.2 (3.5)		25.9 (0.8)	17.8 (2.3)	27.5 (3.1)	49.8 (8.6)	
> 5	13.4 (0.7)	28.3 (2.8)	31.8 (4.1)		13.4 (0.7)	30.1 (3.3)	27 (3.2)	33.4 (7.4)	
Occupation				< 0.001					< 0.001
1	14.6 (0.5)	2.6 (0.8)	0.9 (0.6)		14.6 (0.5)	2.7 (0.9)	1.5 (0.7)	1.1 (1.1)	
2	9.5 (0.4)	2.4 (0.8)	4.1 (1.6)		9.5 (0.4)	4.2 (1.2)	1.7 (0.8)	.	
3	14.5 (0.5)	9 (1.8)	6.5 (2)		14.5 (0.5)	9.2 (2)	8.5 (2.1)	0.8 (0.8)	
4	6.2 (0.7)	24.7 (3)	21.8 (3.7)		6.2 (0.7)	21.8 (3.5)	27 (3.8)	24 (6.5)	
5	11.9 (0.5)	10.3 (1.7)	10.4 (3.3)		11.9 (0.5)	9.6 (1.7)	11.5 (2.8)	10.4 (6.2)	
6	8 (0.3)	14.2 (2)	11.1 (2.2)		8 (0.3)	14.1 (2.4)	12.1 (2.4)	13.5 (4.8)	
7	35.3 (0.6)	36.7 (2.6)	45.2 (4)		35.3 (0.6)	38.5 (3.2)	37.7 (3.7)	50.2 (7.5)	

Numbers are presented as the weighted percentages (SE)

25(OH)D = 25-hydroxyvitamin D; Occupation, 1 = professional; 2 = office workers; 3 = service industry; 4 = agriculture/forestry/fishery; 5 = technician; 6 = laborer; 7 = none (including housewives, students)

**Fig. 2:**
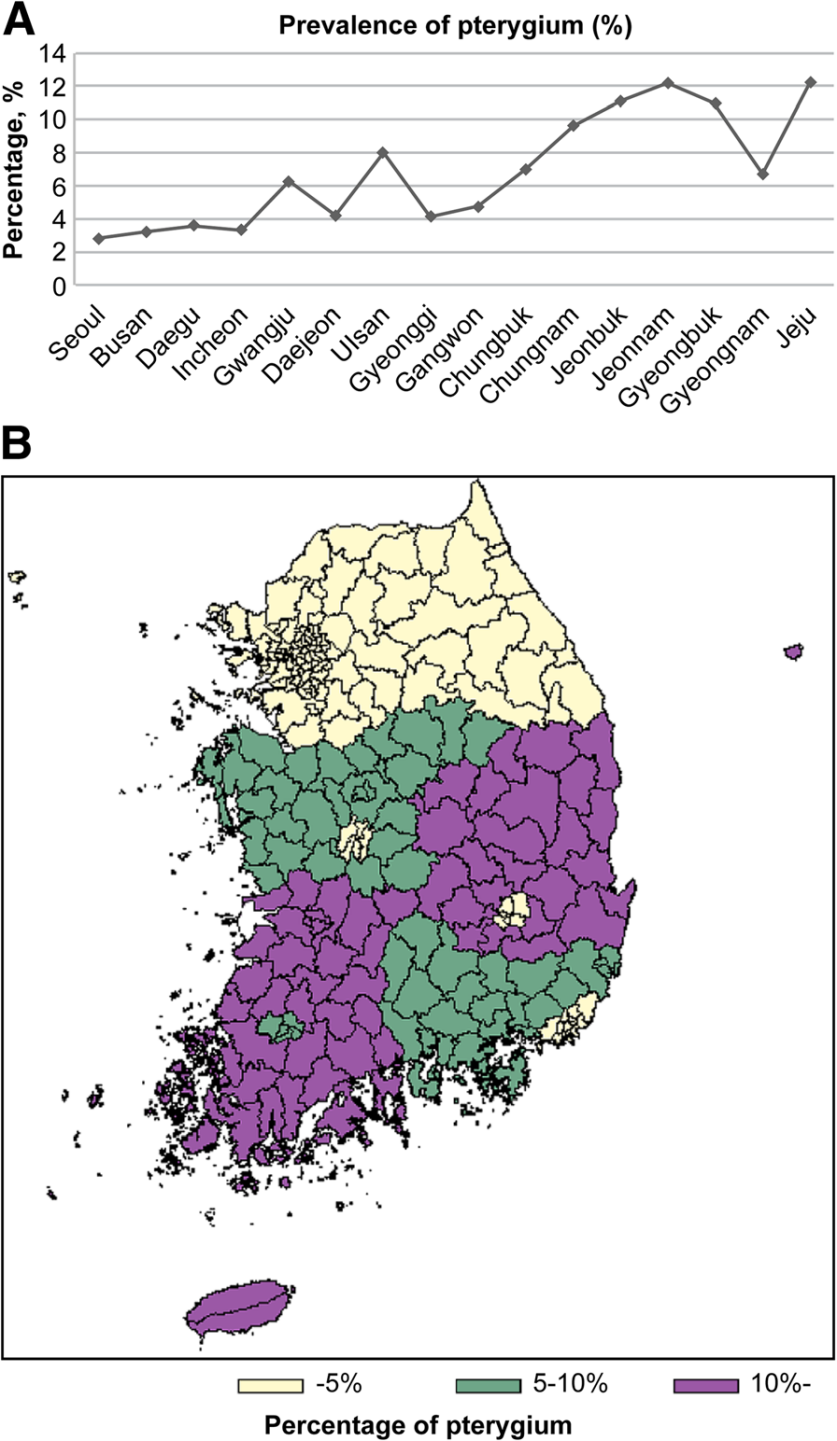
Prevalence of pterygium in South Korea. **a** Prevalence of pterygium stratified by the population of cities. **b** Prevalence of pterygium stratified by spatial distribution. Metropolitan cities: Seoul (*n* = 10,195,318), Busan (*n* = 3,538,484), Daegu (*n* = 2,505,644), Incheon (n = 2,843,981), Gwangju (n = 1,469,216), Daejeon (n = 1,524,583), and Ulsan (n = 1,147,256). Provinces: Gyeonggi (*n* = 12,093,299), Gangwon (n = 1,538,630), Chungbuk (n = 1,565,628), Chungnam (n = 2,028,777), Jeonbuk (n = 1,873,341), Jeonnam (n = 1,909,618), Gyeongbuk (n = 2,698,353), Gyeongnam (n = 3,319,314), and Jeju (*n* = 583,713). The population numbers were obtained from the 2012 census

Figure 2 shows the prevalence of pterygium in South Korea. To investigate whether the urbanization or the latitude of residency was associated with pterygium prevalence, the percentage of pterygium was stratified according to the population of city dwellers and geographical areas. The prevalence of pterygium was negatively associated to latitude of the residential area, but not the size of the cities.

Table 3 shows the ORs (95% CIs) of pterygium according to serum 25(OH)D level. After adjusting age and regular exercise (Model 1), subjects with a serum 25(OH)D level > 30 ng/mL, 25–30 ng/mL, and 15–20 ng/mL had an OR (95% CI) of 1.519 (1.016–2.271), 1.543 (1.097–2.168), 1.819 (1.403–2.357), and 1.553 (1.248–1.933), respectively, compared to those with a serum 25(OH)D level < 15 ng/mL. After further adjustment for BMI, smoking status, and alcohol consumption (Model 2), subjects with a serum 25(OH)D level > 30 ng/mL, 25–30 ng/mL, and 15–20 ng/mL had an OR (95% CI) of 1.515 (1.010–2.274), 1.536 (1.091–2.163), 1.806 (1.393–2.342), and 1.544 (1.238–1.925) compared to those with a serum 25(OH)D level < 15 ng/mL. These associations persisted even after an additional adjustment for diabetes, metabolic syndrome, hypertension, and stress. The *P*-value was < 0.0001 for all comparisons. In the final model, subjects with a daily sun exposure > 5 h had an OR (95% CI) of 1.761 (1.395–2.223) compared to subjects with a daily sun exposure < 2 h. The risk of pterygium was higher in subjects with a serum 25(OH)D level > 15 ng/mL and daily sun exposure > 5 h. Dose-dependent differences were not seen with either serum 25(OH)D level or daily sun exposure.

**Table 3:** Multiple logistic regression analysis for the association between serum 25(OH)D concentration and pterygium prevalence in the Korean population

	Model 1	Model 2	Model 3
Serum 25(OH)D concentration, ng/mL			
> 30	1.519 (1.016–2.271)	1.515 (1.01–2.274)	1.565 (1.035–2.366)
25–30	1.543 (1.097–2.168)	1.536 (1.091–2.163)	1.545 (1.086–2.198)
20–25	1.819 (1.403–2.357)	1.806 (1.393–2.342)	1.8 (1.358–2.386)
15–20	1.553 (1.248–1.933)	1.544 (1.238–1.925)	1.535 (1.216–1.938)
< 15	1	1	1
Daily sun exposure, hours			
> 5	1.683 (1.337–2.117)	1.689 (1.342–2.126)	1.761 (1.395–2.223)
2–5	1.126 (0.889–1.427)	1.126 (0.89–1.425)	1.129 (0.877–1.453)
< 2	1	1	1

25(OH)D = 25-hydroxyvitamin D

Model 1 = adjusted for age and regular exercise

Model 2 = adjusted for Model 1 + body mass index, smoking, and drinking

Model 3 = adjusted for Model 2 + diabetes, metabolic syndrome, hypertension, and stress

## Discussion

Herein, we investigated the prevalence of pterygium according to the serum 25(OH)D level and daily sun exposure. Vitamin D status is assessed by measuring circulating serum 25(OH)D. In this study, we used the serum 25(OH)D level to objectively assess the sun exposure status of each individual. A positive association between serum 25(OH)D levels and pterygium has been shown recently. We used a large population to confirm and further analyze the association between serum 25(OH)D level, daily sun exposure, and pterygium. Our study also showed that a high serum 25(OH)D level was associated with the prevalence of pterygium. Further, the prevalence of pterygium was negatively associated with the residential spatial area of latitude, but not the size of the cities. This significant association remained after adjusting for potential confounding factors such as age, exercise, BMI, smoking, drinking, diabetes, hypertension, metabolic syndrome, and psychological stress. The key finding of the current study was that pterygium prevalence was significantly higher when the subjects were exposed to > 5 h of daily sun and their serum 25(OH)D level was > 15 ng/mL. The clear cut-off level could not be defined because the study was based on survey results. Dose-responsive changes of pterygium percentage according to serum 25(OH)D levels and daily sun exposure were not found, and we assume that changes above a certain threshold would lead to pterygium pathogenesis.

In the human eye, vitamin D-target cells throughout the retina were first identified by the presence of vitamin D-dependent calcium binding protein [15]. Immunohistochemical staining later identified the presence of vitamin D receptors in the epithelium of the cornea, lens, ciliary body, and retinal pigment epithelium, as well as the corneal endothelium, ganglion cell layer, and retinal photoreceptors [22], suggesting that vitamin D is a more ubiquitous molecule in the human eye [23]. Especially, vitamin D is able to reduce inflammatory mediators, enhance barrier function, and protect ocular health [23]. Some previous studies have demonstrated that vitamin D has anti-inflammatory effects at the ocular surface [24, 25]. In previous in vivo studies, topical administration of 25-dihydroxyvitamin D3 inhibited neovascularization and inflammatory cytokines, such as interleukin (IL)-1a and tumor necrosis factor-alpha [26]. In vitro, vitamin D appears to dampen the inflammatory response to infection. In addition, vitamin D augments corneal epithelial barrier function through upregulation of the tight junction proteins occludin and ZO-1 [27]. Therefore, some investigators have suggested that vitamin D has the potential to reverse the harmful effects to the corneal epithelial barrier during infection and can protect against inflammatory conditions [23].

The pathogenesis of pterygium formation is still poorly understood. In previous experimental studies, UV radiation was found to affect the cascade by triggering chronic inflammation [28, 29]. Di Girolamo et al. found that UV induction of proinflammatory cytokines such as IL-6 and IL-8 might play a key role in pterygium development by initiating blood vessel formation, cellular proliferation, tissue invasion, and inflammation [30]. Nakagami et al. showed an increased number of stromal mast cells, which are known to release tumor necrosis factor-alpha, under UV radiation [31]. Kennedy et al. demonstrated increased expressions of IL-1, IL-6, IL-8, and tumor necrosis factor in corneal fibroblasts and corneal epithelium exposed to UVB radiation [32], and Black et al. characterized the inflammatory response of human corneal epithelial cells to UVB (2.5–25 mJ/cm^2^) [33]. UVB causes a dose-dependent increase in the generation of reactive oxygen species in the cells. The authors showed that UVB modulates corneal epithelial cell expression of antioxidants and proinflammatory mediators by distinct mechanisms [33]. Alterations in the expressions of these mediators are likely to be important for regulating inflammation and protecting the cornea from UVB-induced oxidative stress.

In the present study, serum 25(OH)D and sun exposure were also positively associated with recurrence of pterygium. Sunlight is known to affect the outcome of surgery, with chronic conjunctival inflammation seen significantly more often in eyes that are operated on during the summer [34]. Furthermore, Kheirkhah et al. noted that all recurrences were observed in patients with chronic inflammation [35], and concluded that persistent host conjunctival inflammation that is left untreated might lead to a poor surgical outcome. Factors such as dry eye disease and ocular demodicosis may influence pterygium recurrence by causing chronic ocular inflammation.

Our study demonstrated that serum 25(OH)D level was positively associated with the severity of pterygium morphology. Tan et al. suggested that the current simple clinical grading system of pterygium morphology, based on a discrete scale of relative translucency of pterygium tissue, was useful for predicting pterygium recurrence, as shown by the presence of a strong relationship between pterygium recurrence and the initial pterygium morphology [21]. The finding of an association between pterygium morphology and pterygium recurrence herein suggests that a common denominator such as sunlight exposure, represented by serum 25(OH)D, may have implications on the pathophysiological features, cause, and recurrence of pterygium. Our study suggested that individuals with higher serum 25(OH)D levels and who were exposed to more sunlight were more likely to have flesh-type pterygium rather than the atrophic type.

Serum 25(OH)D is the most commonly used measure of vitamin D status, as it is more easily quantified in blood than any other form of vitamin D [36]. Nair-Shalliker et al. provided evidence for a curved linear relationship between solar UV exposure and serum 25(OH)D concentration [12], and Timothy et al. showed a strong association between pterygium and sun exposure after constructing dose-response curves. They obtained the lifetime history of residence, sun exposure patterns, and use of hats, spectacles, and sunglasses via interviews, while measures of potential sun exposure included latitude, daily sunshine hours, and daily global solar radiation energy. The authors showed there were strong positive associations between pterygium and the measures of potential and actual sun exposure. The strongest associations were seen for the estimated daily ocular solar radiation doses, with an OR of 6.9 for the highest quarter of exposure. On the other hand, our study addressed the relationship between individual UVB light exposure and pterygium prevalence using serum 25(OH)D levels. Moreover, as we found that the prevalence of pterygium was negatively associated with the latitude of the residence, the association between sunlight exposure and pterygium became more compelling.

Our study has some limitations. First, the results may be confounded by a lack of data on relevant variables such as information on total time spent outdoors and the season of measurements. Second, the current study has a cross-sectional design, which makes inferring causality difficult. Third, information or recall bias may exist because this study included data from a health interview survey. Forth, evaluation of sunlight exposure was relatively crude. The effect of sunlight exposure may be variable depending on the months of the year and latitude. Further supporting variables related to vitamin D, such as serum parathyroid hormone levels or bone parameters, were not measured. This is the common conflicting issue that vitamin D has a pleiotropic nature, particularly regarding metabolism, and many important adjustments should be considered, including lag time of exposure and serum 25(OH)D level, duration and time of day in sun exposure, and extent of skin surface exposed to the sun or UV radiation. Thus, although this study indicated a possible link between UV exposure, serum 25(OH)D level, and pterygium prevalence, these limitations should be addressed in future studies. Lastly, there is overlapping information between ours and a previous study that investigated the association between serum 25(OH)D levels and pterygium using data from the Korean National and Nutrition Survey [18]. However, there are several differences between the two studies: (i) the period of investigation and the age of the subjects were different; (ii) our study additionally found that the prevalence of pterygium was associated with the latitude of residence, and a crude cut-off value of sun exposure duration and serum 25(OH)D levels for the risk of pterygium was suggested; and (iii) our study also evaluated the association between serum 25(OH)D levels and pterygium recurrence and morphology. Nonetheless, despite these limitations, the major strength of this study is the relatively large number of participants and the study design using systemic stratified, multistage, clustered, random sampling methods. Moreover, as Koreans have relatively uniform genetic and environmental influences, including a single race, climate, and food culture, the results may be more consistent than those of other previous population-based studies.

## Conclusion

In conclusion, the present study provides epidemiological evidence of an association between serum 25(OH)D level and pterygium in a representative Korean population. Our results suggest that the serum 25(OH)D level is positively correlated with the prevalence of pterygium. This might suggest that although serum 25(OH)D exerts an anti-inflammatory action, its effect on the ocular surface is minimal and not enough to prevent local UV insult on the conjunctiva. Rather, our study results support the strong association between pterygium and sunlight exposure using objectively measured serum 25(OH) levels.

## Abbreviations

25(OH)D: 25-hydroxyvitamin D
BMI: Body mass index
CI: Confidence interval
IL: Interleukin
KNHANES: Korea National Health and Nutrition Examination Survey
OR: Odds ratio
UVB: Ultraviolet-B
WC: Waist circumference
